# Can Different Dietary Protein Sources Influence the Survival, Growth, and Physiology of 0^+^Marron (*Cherax cainii*) Exposed to Feed Deprivation?

**DOI:** 10.3390/ani14243591

**Published:** 2024-12-12

**Authors:** Thi Thanh Thuy Dao, Ravi Fotedar

**Affiliations:** 1 School of Molecular and Life Sciences, Curtin University, 1 Turner Avenue, Bentley, WA 6102, Australia; t.dao1@postgrad.curtin.edu.au; 2 Research Institute for Aquaculture No. 3 (RIA3), 02 Dang Tat Street, Nha Trang 650000, Vietnam

**Keywords:** feed deprivation, immunity, physiology, protein sources, *Cherax cainii*

## Abstract

We investigated the effect of feed deprivation for 45 days on the growth, immunity, and health of 0^+^marron (*Cherax cainii*) initially fed for 110 days on various protein sources including fishmeal (FM), poultry by-product meal (PBM), black soldier fly meal (BSFM), soybean meal (SBM), lupin meal (LM), and tuna hydrolysate. The marron were weighed and sacrificed immediately after feeding stopped (day 0) and at days 15, 30, and 45 after the feed deprivation trial commenced. Total haemolymph count, differential haemocyte count, lysozyme activity, protease activity, total bacterial count in the digestive tract, and organosomatic indices were analysed. Initially feeding marron any protein sources did not influence the percentage of weight gain and specific growth rates of marron. All marron showed more than 83% survival; however, marron fed soybean meal showed significantly lower survival than others. Dietary sources of protein altered organosomatic indices of starved marron during various starvation periods and resulted in a significant decrease in total haemocyte counts, lysozyme activity, protease activity, and bacterial count in the digestive tract of marron. Starved marron initially fed PBM and BSFM showed higher tolerance to starvation, followed by marron initially fed FM and SBM, while marron initially fed TH and LM showed the highest susceptibility to starvation.

## Simple Summary

Protein sources can influence how marron, the largest farmed freshwater crayfish, can combat stress caused by an absence of food. Marron were fed different protein sources for 110 days and then starved for 45 days while being housed in individual compartments. Marron immunity was determined by analysing their haemolymph, gut bacteria, and protein-digesting enzymes, whereas health status was determined by taking body tissues like hepatopancreas and tail muscles and determining the moisture levels in them. The results showed that marron fed protein sourced from animal origin were healthier and more tolerant to starvation than those fed lupin meal. No marron showed any significant increases in growth rates during the period of starvation, whereas the survival of all starved marron was more than 83%. However, the soybean protein diet resulted in the significantly lowest survival. Animal-based protein sources, except for tuna hydrolysate, resulted in higher immunity of the starved marron than plant-sourced protein. We postulated that the presence of chitin in black soldier fly larval meal may have contributed to enhancing the immune competence in marron.

## 1. Introduction

Marron (*Cherax cainii*) is the world’s largest farmed freshwater crayfish species and is endemic to Western Australia. In wild habitats, the species frequently experiences shortages in food supply and thus needs to cope with extended periods of feed deprivation. During the feed deprivation phase, like that of other crayfish species [1], the metabolism of marron could be adapted to utilise stored energy in the hepatopancreas and tail muscles, hence altering organosomatic indices, to survive and maintain basic body functions. For example, the crayfish may experience a decrease in the hepatopancreas weight and functionality as stored reserves are gradually depleted to meet metabolic demands. Jones and Obst [2] reported a reduction in the wet and dry hepatosomatic indices (Hiw and Hid) and increased hepatopancreas moisture (HM) in yabbies (*C. destructor*) starved for 16 weeks. Starved red claw crayfish (*C. quadricarinatus*) had a lower hepatosomatic index [3]. This adaptive response reveals the intricate relationship between nutrient availability, physiological adaptations, and survival strategies employed by crayfish species to endure feed deprivation conditions. Past research has shown that organosomatic indices can be used as health indicators in marron [4,5,6].

The characterisation of the haemolymph and a differential haemocyte analysis are also useful tools for health estimation in animals [7] and can be applied to marron for health [8] during starvation periods. Marron rely on their innate immunity to protect them against pathogens. The haemocytes play a major role in the innate immune defence system by eliminating foreign particles that penetrate the haemocoel through phagocytosis and aiding wound healing. For example, marron did not reduce its growth rate, but lysozyme activity and THC decreased significantly after four weeks of starvation [9]. The decrease in THC in the haemolymph can also be attributed to the presence of a pathogen or environmental stress [10].

Some of the immune indices such as the activities of superoxide dismutase, catalase, acid phosphatase, and lysozyme are also affected during feed deprivation periods. Studies on red claw crayfish and red swamp crawfish (*Procambarus clarkii*) during starvation periods showed decreased glutathione levels and activities of superoxide dismutase and lysozyme [3,11]. Similarly, white-leg shrimp (*Litopenaeus vannamei*) showed decreased hyaline cells (HCs), granular cells (GCs), and total haemocyte count (THC) when starved for 14 days [12,13]. Starvation in red swamp crawfish and red claw crayfish has been shown to significantly impact the activities of trypsin, lipase, and amylase [3,14,15]. These changes in enzyme activities highlight their ability to adjust their immunity to cope with limited food availability and allow them to potentially increase their survival during periods of feed deprivation.

The capacity of crayfish to endure long periods of starvation has been studied and reported by [2,15,16,17,18]. However, there no comparative physiological analysis has been performed on how feeding marron on various dietary sources of protein can assist marron in combating the stress caused if they are exposed to prolonged feed deprivation later during their life cycles. Currently, some of the protein sources used in the diets of marron and other farmed species include black soldier fly (*Hermetia illucens*) larvae (BSFL) [19,20], poultry by-product meal (PBM) [21,22], fish protein hydrolysate (FPH) [23,24], and plant proteins, such as soybean meal (SBM) [25,26,27,28] and lupin meal (LM) [29,30].

BSFL has generated interest as an alternative to fishmeal because of its rapid reproduction cycle, substantial biomass yield, and efficient conversion of proteins and lipids [31]. Studies of the successful utilisation of BSFL have been found for white-leg shrimp [32,33,34] and marron [19,35]. PBM has been successfully utilised in aquafeeds for freshwater crayfish and shrimp species, including red claw crayfish [21,36], North American signal crayfish (*Pacifastacus leniusculus*) [37], marron [19,38], red swamp crawfish [22], giant freshwater prawn (*Macrobrachium rosenbergii*) [39], and oriental river prawn (*M. nipponense*) [40]. FPH is a highly nutritious product derived from fish by-products. According to Ospina-Salazar et al. [41], FPH is characterised by well-balanced and biologically active immunomodulating low-molecular-weight peptides, which have the potential to act as an attractant and enhance palatability [42]. FPH has been used as a supplement in many aquaculture diets to improve immunity [23,24,43].

The use of plant proteins, such as SBM and LM, as dietary proteins was previously studied in yabbies [26], North American signal crayfish [25], and red swamp crawfish [27]. However, using plant protein sources has some limitations which include imbalanced methionine, lysine, and tryptophan, the presence of anti-nutritional factors (ANFs) including phytate and trypsin inhibitors, and low palatability [44,45,46] that can negatively impact growth.

Several studies have reported feeding dietary supplements to marron to improve immunity to pathogen diseases and environmental stressors [8,47,48,49]; however, there is no information available on whether different diets can have varied impacts on the physiological status and/or efficiencies in combating chronic stress caused by the prolonged absence of food. There is limited published information on the relationship between various protein diets and tolerance to starvation in any crayfish, including marron, except for limited research by Foysal et al. [9], who studied the effects on gut microbial characterisation. Consequently, this study aims to assess the starvation tolerance of marron initially fed various protein diets, while also determining immune parameters and physiological responses during the period of feed deprivation.

## 2. Materials and Methods

### 2.1. Formulated Feed Used Before the Feed Deprivation Trial Commenced

All dry ingredients of the diets were purchased from Specialty Feeds Company, Glen Forrest, Western Australia. The BSFL were supplied by Future Green Solutions, Western Australia. BSFL were subjected to dehydration in an oven for 48 h at 60 °C. Subsequently, BSFL were finely ground into a powder using a coffee grinder (Sunbeam Café Series, EMM0500BK, China). Southern bluefin tuna *Thunnus maccoyii* hydrolysate (TH) was provided by SAMPI, Port Lincoln, Australia. The feed formulations and proximate composition are presented in Table 1. The amino acid profiles of the test diet are given in Table 2. The same feed formulation and amino acids of experimental diets were used as in the previous studies by Foysal et al. [35] and Dao et al. [50].

### 2.2. Feed Deprivation Experimental Design

The feed deprivation trials were carried out in Curtin Aquatic Research Laboratory (CARL) at Technology Park, Bentley Western Australia, 6102. Marron were purchased from Blue Ridge Marron, Manjimup, Western Australia, and transported to CARL. After two weeks of acclimation, the marron were randomly stocked into 18 tanks (six dietary treatments with three replications per treatment). Each tank had a capacity of approximately 300 L, a diameter of 100 cm, and a height of 40 cm. The tanks were fitted with an external canister filter, Astro^®^ AS-2212, [DoPhin, Qingdao, Shandong, China] that provided continuous aeration. Marron were housed individually in labelled 1000 mL plastic containers (150 × 110 × 70 mm) to avoid cannibalism and to facilitate the collection of individual moult-related data. Containers had small holes to ensure water exchange with the main tank. Marron were fed with six various protein source diets for 110 days. At the end of the feeding trials, the physiological responses of marron in the trial were evaluated as per our previous research [50], before the marron were starved for 45 days. The data collected at the end of the feeding trial were considered as data collected on day 0 of the current starvation trial.

Following the feeding trial, the 10 random marron from each tank (three replicated tanks per dietary treatment) continued to go through the current feed deprivation trial. No food was given to any marron for 45 days. Water exchange was conducted every two weeks at a rate of 50% of the total water volume to ensure there was no visible planktonic or detritus presence in the culture system. Water quality parameters, including dissolved oxygen (DO), pH, temperature, and total ammonia, were assessed once a week using the multiparameter meter (YSI, Yellow Springs, OH, USA) and an API ammonia NH_3_/NH_4_ kit (API Aquarium Pharmaceuticals™, Mars, Inc., Chalfont, PA, USA). The natural photocycle of the marron was reversed by maintaining 12 h of darkness during the day using artificial light in the dark laboratory. During the experiment, the mortality and weight of the moulted marron were recorded. The treatment names are depicted in Table 3.

### 2.3. Sample Collection and Parameter Analysis

The marron samples were conducted at days 0 (immediately after the feeding was terminated), 15, 30, and 45 of the feed deprivation periods. One random marron per tank was selected, and haemolymph was withdrawn into a 1 mL sterile syringe (27-gauge) containing anticoagulant (haemolymph/anticoagulant = 1:1) [51]. The hepatopancreas and the digestive tract were removed and collected for digestive protease activity and total gut bacterial count. The haemocyte parameters including THC and differential haemocyte count (DHC) were conducted and calculated using descriptions provided by Sang et al. [8]. The lysozyme activity of marron haemolymph was determined by the turbidimetric assay, as described by Tulsankar et al. [51].

The hepatopancreas were removed and stored at −80 °C for the protease assay. A sample of 0.3 g hepatopancreas was weighed and homogenised in 3 mL of phosphate-buffered saline (PBS). The mixture samples were centrifuged at 10,000× *g* at 4 °C for 10 min. After centrifuging, the supernatant was used for protease activity measurement, and tissue debris at the bottom was discarded. A commercial kit (Thermo Scientific TM Pierce TM Protease Assay Kit, Adelaide, Australia) was used to determine the protease activity in hepatopancreas. The protease activity was measured at the absorbance of 450 nm using an MS212 reader (Titertek Plus, Tecan, Grodig, Austria), as outlined by Dao et al. [50].

Total bacteria in the gut were determined following the protocols of [52] with some modifications. The gut was removed from the marron in each tank and transferred into a sterilised Eppendorf tube. The gut was weighed to four decimal places and homogenised with PBS, pH 7.4, using a glass homogeniser. It was further serially diluted using PBS up to 10^−7^ dilutions. A 100 µL sample from three dilutions was selected and spread on a Tryptone glucose yeast agar plate (Oxoid LTD, Basingstoke, Hampshire, England). The plates were incubated for 48 h at 30 °C. Colonies were counted for each plate and expressed as colony-forming units (CFUs). The total number of bacteria per gram of sample was obtained by multiplying the colony count by the dilution factor.

Another marron per tank was randomly collected and sacrificed, and hepatopancreas and marron tail muscles were removed to calculate organosomatic indices as indicators of health, as described and followed by Fotedar [5]. Hepatopancreas moisture content (HM), wet and dry hepatosomatic indices (Hiw and Hid), tail moisture content (TM), and wet and dry tail muscle indices (Tiw and Tid) were calculated following formulas.

$$\mathrm{HM}=100\times \frac{(\mathrm{Weight}\;\mathrm{of}\;\mathrm{wet}\;\mathrm{hepatopancreas}-\mathrm{Weight}\;\mathrm{of}\;\mathrm{dry}\;\mathrm{hepatopancreas})}{\mathrm{Weight}\;\mathrm{of}\;\mathrm{wet}\;\mathrm{hepatopancreas}}$$

$$\mathrm{TM}=100\times \frac{(\mathrm{Weight}\;\mathrm{of}\;\mathrm{wet}\;\mathrm{tail}\;\mathrm{muscle}-\mathrm{Weight}\;\mathrm{of}\;\mathrm{dry}\;\mathrm{tail}\;\mathrm{muscle})}{\mathrm{Weight}\;\mathrm{of}\;\mathrm{wet}\;\mathrm{tail}\;\mathrm{muscle}}$$

$$\mathrm{Hiw}=100\times \frac{\mathrm{Weight}\;\mathrm{of}\;\mathrm{wet}\;\mathrm{hepatopancreas}}{\mathrm{Total}\;\mathrm{weight}\;\mathrm{of}\;\mathrm{maron}\;}$$

$$\mathrm{Tiw}=100\times \frac{\mathrm{Weight}\;\mathrm{of}\;\mathrm{wet}\;\mathrm{tail}\;\mathrm{muscle}}{\mathrm{Total}\;\mathrm{weight}\;\mathrm{of}\;\mathrm{maron}\;}$$

$$\mathrm{Hid}=100\times \frac{\mathrm{Weight}\;\mathrm{of}\;\mathrm{dry}\;\mathrm{hepatopancreas}}{\mathrm{Total}\;\mathrm{weight}\;\mathrm{of}\;\mathrm{maron}\;}$$

$$\mathrm{Tid}=100\times \frac{\mathrm{Weight}\;\mathrm{of}\;\mathrm{dry}\;\mathrm{tail}\;\mathrm{muscle}}{\mathrm{Total}\;\mathrm{weight}\;\mathrm{of}\;\mathrm{maron}\;}$$

### 2.4. Calculations

$$\mathrm{Weight}\;\mathrm{gain}\;\mathrm{WG}\;(\%)=100\;\times \frac{\left(\mathrm{Final}\;\mathrm{weight}\;-\;\mathrm{Initial}\;\mathrm{weight}\right)}{\mathrm{Initial}\;\mathrm{weight}\;}$$

$$\mathrm{Specific}\;\mathrm{growth}\;\mathrm{rate}\;\mathrm{SGR}\;(\%/\mathrm{day})=100\times \frac{\left(\ln\;\mathrm{Final}\;\mathrm{weight}\;-\;\ln\;\mathrm{Initial}\;\mathrm{weight}\right)}{\mathrm{Number}\;\mathrm{days}\;}$$

$$\mathrm{Survival}\;\mathrm{rate}\;(\%)=100\times \frac{\mathrm{Final}\;\mathrm{numbers}\;\mathrm{of}\;\mathrm{marron}}{\mathrm{Initial}\;\mathrm{numbers}\;\mathrm{of}\;\mathrm{marron}\;}$$

$$\mathrm{Moulting}\;\mathrm{rate}\;(\%)=100\times \frac{\mathrm{Numbers}\;\mathrm{of}\;\mathrm{moulted}\;\mathrm{marron}\;}{\mathrm{Total}\;\mathrm{numbers}\;\mathrm{of}\;\mathrm{maron}\;}$$

### 2.5. Statistical Analysis

SPSS version 25.0 was used to analyse the data. Results were presented as mean ± SE. The Shapiro–Wilk test and the Levene test assessed the normality and homogeneity of data before analysis. One-way ANOVA analysis was used examine to growth performance and moulting rate. The cumulative percentage survival of feed deprivation was calculated using Kaplan–Meier curves and the Log-rank test (Mantel–Cox). Two-way ANOVA analysis followed by Tukey post hoc test for multiple comparisons at the significant difference *p* < 0.05 was performed on immune parameters and organosomatic indices. A pair *t*-test was used to determine the significant difference between before and after protease activity and bacterial count in the gut of marron with *p* < 0.05.

## 3. Results

### 3.1. Growth Performance and Survival

**Figure 1:**
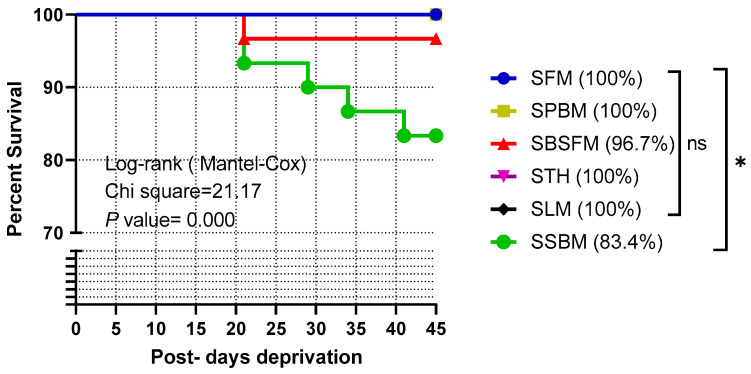
The survival rate of the marron fed test diet during the starvation test. Mean ± SE (n = 3). ns indicates not significant. * *p* < 0.05 denotes significant differences. SFM: starved marron initially fed fishmeal; SPBM: starved marron initially fed poultry by-product meal; SBSFM: starved marron fed initially black soldier fly meal; STH: starved marron initially fed tuna hydrolysate; SLM: starved marron initially fed lupin meal; SSBM: starved marron initially fed soybean meal.

Feed deprivation did not result in any significant (*p* > 0.05) changes in the growth performance in terms of WG%, SGR%/day, total moults, and moulting rate% of marron (Table 4) and were in the range of 4.58 to 9.56%, 0.09 to 0.18%/day, 3 to 4, and 10 to 13%, respectively. Kaplan–Meier analysis revealed significant differences in the survival rate of marron among groups (χ^2^ = 21.17, df = 5, *p* = 0.000). The survival rate of the starved marron, initially fed SBM, was 83.4%, which was significantly lower than marron initially fed the rest of the diets (Figure 1).

### 3.2. Immune Responses

**Figure 2:**
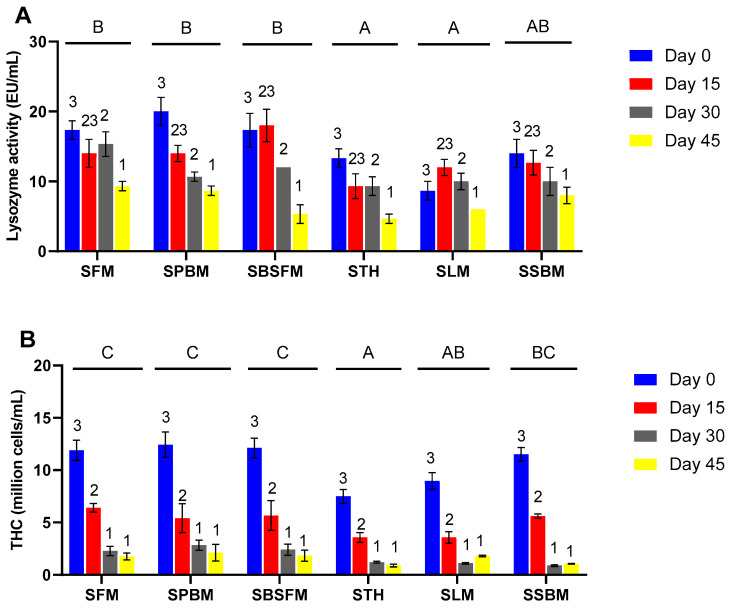
Lysozyme activity (**A**) and THC (**B**) in marron during feed deprivation. The values are mean ± SE (n = 3). Letters (A, B, C) indicate significantly different means for different groups at *p* < 0.05. Different numbers (1, 2, 3) denote significantly different means at times of feed deprivation. Two-way ANOVA, followed by Tukey post hoc test at *p* < 0.05 determined the effects of treatments on lysozyme, feed deprivation durations on lysozyme activity, and their interaction between treatments and feed deprivation durations. SFM: starved marron initially fed fishmeal; SPBM: starved marron initially fed poultry by-product meal; SBSFM: starved marron fed initially black soldier fly meal; STH: starved marron initially fed tuna hydrolysate; SLM: starved marron initially fed lupin meal; SSBM: starved marron initially fed soybean meal.

Lysozyme activity was influenced by initial feeding (treatments) various protein sources and the feed deprivation durations. SFM, SPBM, and SBSFM exhibited significantly higher (*p* < 0.05) lysozyme activity than STH and SLM. Lysozyme activity was similar until day 30 but dropped significantly and progressively as the starvation extended to 45 days (Figure 2A). Similarly, a declining trend in THC during the starvation period was observed among marron initially fed various diets (Figure 2B). THC values were similar at day 30 and day 45. Starved marron initially fed TH showed the lowest THC, followed by SLM and SSBM, while the highest THC was observed in SFM, SPBM, and SBSFM.

**Figure 3:**
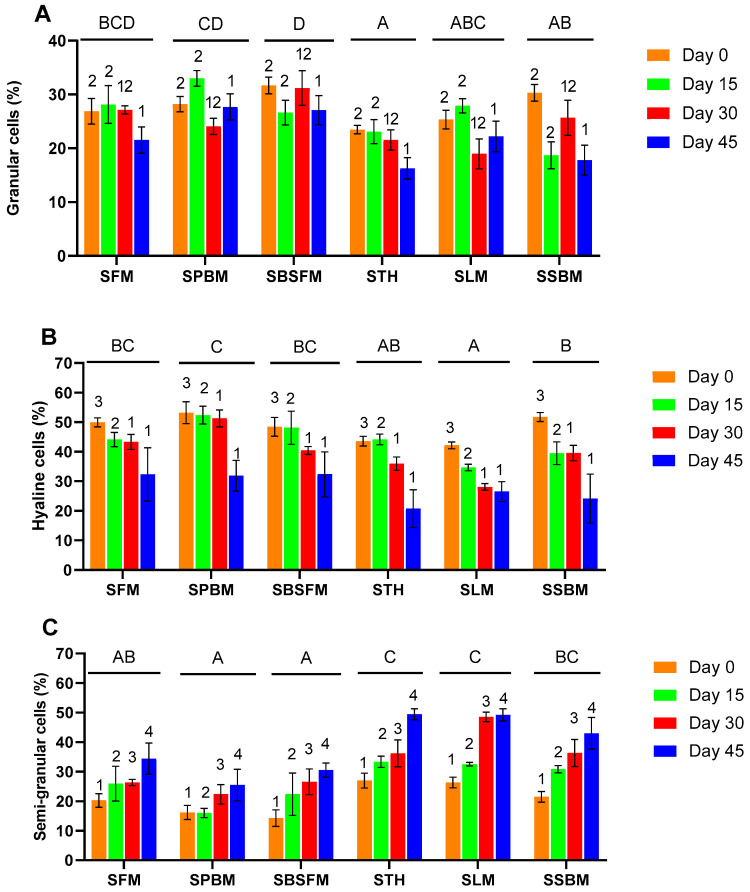
Differential haemocyte count of starved marron during feed deprivation period. Granular cells (**A**), hyaline cells (**B**), and semi-granular cells (**C**). The values are mean ± SE (n = 3). Letters (A, B, C, D) indicate significantly different means for different groups at *p* < 0.05. Different numbers (1, 2, 3, 4) denote significantly different means at different times of feed deprivation. Two-way ANOVA, followed by Tukey post hoc test at *p* < 0.05 determined the effects of treatments on lysozyme, feed deprivation durations on lysozyme activity, and their interaction between treatments and feed deprivation durations. SFM: starved marron initially fed fishmeal; SPBM: starved marron initially fed poultry by-product meal; SBSFM: starved marron fed initially black soldier fly meal; STH: starved marron initially fed tuna hydrolysate; SLM: starved marron initially fed lupin meal; SSBM: starved marron initially fed soybean meal.

The percentage of granular cells in starved marron was significantly lower at day 45 than in the first 30 days of feed deprivation. SBSFM and SPBM showed a significantly (*p* < 0.05) higher percentage of granular cells than the other marron (Figure 3A). The hyaline cell proportion of starved marron significantly decreased from day 0 to day 30, but the same percentages of hyaline cells were found on day 30 and day 45 (Figure 3B). By contrast, the semi-granular cell percentages of starved marron increased significantly (*p* < 0.05) as the starvation time progressed (Figure 3C). STH and SLM obtained higher rates of semi-granular cells than other starved marron.

**Figure 4:**
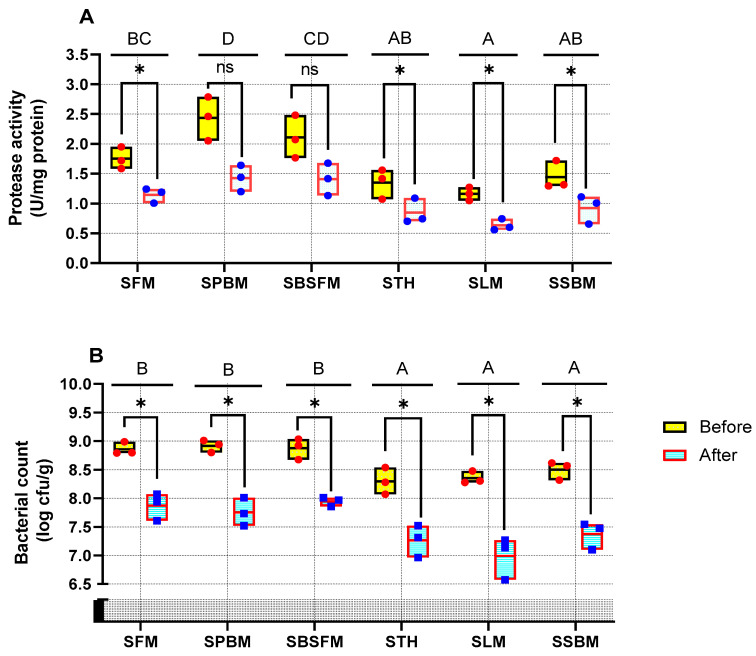
The protease activity (**A**) and total bacterial count (**B**) of starved marron before and after feed deprivation. Two-way ANOVA, followed by Tukey post hoc test with *p* < 0.05 determined the effects of treatments on lysozyme, feed deprivation durations on lysozyme activity, and their interaction between treatments and feed deprivation durations. Letters (A, B, C, D) represent significant differences among treatments. * *p* < 0.05 indicates a significant difference before and after feed deprivation. ns denotes non-significant differences. A paired *t*-test determined the significant difference between the starved marron groups before and after feed deprivation. The line within each box represents the median. The results are expressed in mean ± SE (n = 3). The dots indicate the replicate (n).

Except for SPBM and SBSFM, feed deprivation led to a significant (*p* < 0.05) decrease in protease activity in all marron (Figure 4A). STH, SLM, and SSBM had significantly lower (*p* < 0.05) protease activity, while SPBM and SBSFM had the highest protease activity. The total bacterial count in the gut of starved marron also showed a similar trend in reduction among starved group marron (Figure 4B). SFM, SPBM, and SBSFM exhibited significantly higher total bacterial count in the gut (*p* < 0.05) than STH, SLM, and SSBM.

### 3.3. Organosomatic Indices

Different organosomatic indices of feed-deprived marron as tools to measure marron health, in response to various animal- and plant-based diets are presented in Table 5. Feed deprivation significantly influenced the HM%, TM%, Hiw, Hid, Tiw, and Tid.

Hepatopancreas and tail muscle moisture levels of marron significantly increased between day 0 and day 15 of feed deprivation, increasing by approximately 59–80% and 79–83%, respectively; afterward, no significant differences were observed. The highest HM% was found in STH and SLM, and significantly lower HM% was in SBSFM and SFM. There were no significant (*p* > 0.05) differences in the TM% between the six marron dietary groups.

The Hiw and Hid significantly decreased (*p* < 0.05) as the starvation period progressed. Hiw of SLM was significantly lower than that in other starved marron dietary groups. The SBSFM had the highest Hid compared to the other marron, while the lowest Hid was observed in STH.

Initially feeding various diets did not influence Tiw and Tid in starved marron during feed deprivation. Tiw among starved marron was significantly lower at day 45 than day 0. The Tid values significantly decreased on day 15 compared to day 0; however, there were no changes in Tid after 30 and 45 days.

## 4. Discussion

Crustaceans can frequently be exposed to various periods of food absence, mainly the absence of macronutrients that otherwise are the source of energy, leading to a reduction in growth or no growth. Most likely, there is a higher probability that crustaceans have more access to micronutrients available in their aquatic environment due to their feeding habits. Feed deprivation can lead to physiological alterations including biomechanical changes, ultimately influencing growth and survival. Under experimental conditions, these physiological changes can easily be measured and may be used to predict future survival, growth, and health including the ability to fight any environmental stressors. Hence, it is important to investigate how feed deprivation affects the health and immunity of freshwater crayfish, including marron under controlled experimental conditions. However, there is a possibility that if marron were eating different protein sources before they were exposed to feed deprivation conditions, their physiological response to starvation may vary. The variation in the physiology of starved marron could be because different proteins, having different amino acids and a variety of structures, may have either different storage efficiencies or provide marron with different physiological advantages to combat any stress caused by future malnutrition. Therefore, this study aimed to evaluate and compare the physiology of starved marron initially fed on different protein types.

In the current study, feed deprivation did not influence the growth performance of starved marron initially fed different protein sources. All marron showed positive WG and SGR at the end of the feed deprivation period indicating that the energy produced during the pre-starvation feeding was efficiently stored and converted into body weight during the absence of feeding, or there were enough nutrients present in the rearing system due to the primary productivity, although all attempts were made to minimise it, that was converted to body mass. In the present study, the SGR of starved marron ranged from 0.09 to 0.18%/day, which is lower than the findings of Foysal et al. [9] who achieved SGR values of 0.41%/day during 4 weeks of starvation. This could be attributed to there being less natural food in the form of primary productivity present in the current trial and/or longer study period. The inconsistent results may also be due to a significant difference in the weight of the marron, as the present study used 3 g juvenile 0^+^marron, whereas Foysal et al. [9] used 69 g adult marron.

Marron postponed the moult during feed deprivation, as the current results reported a low 10–13% moulting rate, which agrees with previous studies on red claw crayfish. Feed deprivation affected the moult numbers [16,53], as no moulting occurred in red claw crayfish when no food was provided in a control treatment. Similarly, Sacristán et al. [3] reported the number of moults significantly lowered in starved red claw crayfish than in fed crayfish during 80 days of starvation. A study of white-leg shrimp found that long-term fasting decreased SGR and survival rate [54]. This can be stated that during the starvation period, not enough energy is directed to the moulting process.

In the present study, a high survival rate that ranged from 83% to 100% was achieved, which is in agreement with Stumpf and López Greco [55], who reported a high survival rate of red claw crayfish over 90% across all treatments at the end of food restriction. However, Osman et al. [56] reported that red swamp crawfish showed decreased survival rates due to starvation and aggressive behaviour, emphasising that the negative impact of prolonged starvation on crayfish survival may be related to the changed behaviour. The survival rate gradually decreased with the increase in starvation time when starved marron were initially fed on SBM and attained the lowest level among all other marron groups. It is likely that if the feed deprivation period was extended, further mortalities could have occurred.

Feed deprivation results in reduced physiological and immune status in white-leg shrimp [13], similar to the current study. The granular cells and hyaline cells of the starved marron in each dietary treatment decreased with the increased time of feed deprivation, whereas semi-granular cells showed the opposite trend, increasing over time. Starved marron initially fed LM exhibited significantly lower hyaline cell counts than those starved marron initially fed FM, PBM, and BSFM, which is consistent with Saputra et al. [57], who reported the dietary inclusion of LM led to a lower percentage of hyaline cells following exposure to high temperature. However, the higher percentage of granular cells and hyaline cells in starved marron initially fed FM, PBM, and BSFM suggested that a weak nutritional condition can limit the organism’s ability to physiological compensation during periods of food deprivation. This indicates that the type of protein ingredients plays a crucial role in how marron responds to nutritional stress.

The immune responses, such as lysozyme activity and THC, exhibited a drop in their levels during the period of feed deprivation. Foysal et al. [9] reported similar results, showing a significant decrease in lysozyme, THC, and immune-relevant pro- and anti-inflammatory cytokines in marron following four weeks of starvation. The starved marron initially fed FM, PBM, and BSFM exhibited higher THC levels than those previously fed TH and LM. This suggests that the starved marron that were initially fed animal protein, except TH protein, may have a better tolerance than those initially fed plant protein dietary sources. On day 30, the decrease in lysozyme and THC in starved marron signalled the worst immunity after four weeks of starvation. This is consistent with research by Jussila et al. [58], who looked at the relationship between THC and the physical health of western rock lobster (*Panulirus cygnus*) and found that stressed-out and moribundly obese lobsters had much lower THC levels than healthy lobsters.

Chen et al. [59] showed that dietary BSFM enhanced intestinal immunity and microbiota in white-leg shrimp improving resistance to *Vibrio* infection. A study on white-leg shrimp by Pascual et al. [60] demonstrated that dietary protein levels can govern the immune condition of shrimp, for example, shrimp fed a diet containing 40% protein levels can tolerate immune and physiological suppression for 14 days, whereas shrimp fed 5% dietary protein can tolerate 7 days of food deprivation. Additionally, the dietary protein levels affected how crayfish responded immunologically to starvation. As an example, Zenteno-Savín et al. [61] found that starved red claw crayfish had less oxidative damage and lipid peroxidation when they were fed a higher protein diet (31–36%), indicating that protein in the diet protected against the negative immune effects of starvation.

The initial feeding of different dietary protein sources influenced the protease activity in starved marron during feed deprivation. In the present research, protease activity at post-feed deprivation was lower than at the commencement of starvation across all treatments, except for SPBM and SBFM. This could be explained by the depression of digestive activity being able to attenuate the utilisation of energy reserves, thereby leading to an adaptative strategy to deal with food-deprived conditions and to prevent damage or death [62]. Similarly, a significant decrease in protease activity during periods of starvation was observed in white-leg shrimp [63] and Chinese white shrimp (*Fenneropenaeus chinensis*) [64]. Protease enzymes could be affected by food quality and the absence of food [65,66,67]. Starved marron initially fed PBM and BSFM exhibited significantly higher protease activity compared to those fed SBM, LM, and TH at the end of the feed deprivation period due to the presence of ANFs (such as trypsin inhibitors and phytates) in plant-based proteins reducing digestion [46,68]. A previous study confirmed that a marron fed LM diet produced lower protease enzymes than marron fed animal protein diets [50]. Additionally, the lower protease activity could be due to the absence of food movement in the gut, resulting in low gut peristalsis and lower stimulation of the marron’s entire digestive tract, thereby decreasing the secretion of digestive enzymes [65,66].

It has been shown that feed deprivation can reduce the bacterial diversity in the gut of starved marron [9], similar to the present study where the total bacterial count in the gut was significantly lower in feed-deprived marron than the same marron who were fed at the commencement of the trial. Changes in the gut bacterial community directly influence digestive and immune enzyme activities, thereby influencing the growth traits of white-leg shrimp, as reported by Dai et al. [69]. At the end of the trial, SFM, SPBM, and SBSFM exhibited higher bacterial counts than marron who were initially fed SBM, LM, and TH, indicating starved marron initially fed animal protein diets, except for those fed TH, had higher total bacterial counts than the starved marron initially fed plant protein diets. The results are supported by Foysal et al. [35], who reported that plant protein sources (LM and SBM) showed significantly lower bacterial numbers in the marron gut compared to PBM and BSFM, but not a significant difference with FM and TH that resulted in lower bacterial count in the gut. Chitin in BSFM serves as a preferred substrate for *Lactobacillus* growth, thus aiding in the increase in bacterial counts. Furthermore, bacterial communities may relate to immune responses and digestive enzymes as opportunistic pathogens like *Vibrio* spp. are more abundant during starvation, which is the reason starved marron are more susceptible to pathogen infections.

The feed deprivation reduced the health status of the marron as every starved marron group exhibited a decrease in hepatopancreas moisture and hepatosomatic indices at day 45 compared to day 0 of feed deprivation. In contrast, a previous study on marron fed different protein sources found no significant differences in health, indicated by organosomatic indices before and after high-temperature exposure [57]. The inconsistent results may be due to the short experimental period (5 days) when compared to our 45-day starvation challenge and the higher influence caused by the quality of protein rather than environmental stressors like temperature. Sacristán et al. [3] reported that the hepatosomatic index value decreased at day 15 in starved red claw crayfish and that after starvation, the Hiw value was significantly lower than that in fed crayfish. Similarly, Calvo et al. [70] found that Hiw and Hid were significantly lower in starved juvenile red claw crayfish than fed juvenile crayfish at days 40 and 50 of starvation. When food was provided, Hiw and Hid values went up in the starved red claw crayfish and were the same as those in the control group. The marron fed TH and LM initially exhibited higher HM and lower Hiw and Hid than other starved marron; hence, STH and SLM showed more susceptibility than SFM, SPBM, SBSFM, and SSBM. Therefore, it can be suggested that the marron initially fed TH and LM were in poor physiological condition [71], and starving them led to further deterioration of their health.

## 5. Conclusions

Starved marron initially fed PBM and BSFM showed high tolerance, followed by staved marron initially fed FM and SBM, while marron initially fed TH and LM were more susceptible to starvation. Except for the SBM diet, feed deprivation did not alter the survival rate but influenced the immune responses, digestive protease enzyme, bacterial count, and health indicated by organosomatic indices. Lysozyme and total haemocyte count decreased significantly during the nutrient deprivation period. Protease activity and bacterial counts in starved marron were significantly lower between pre- and post-feed deprivation. Finally, the marron are well adapted to tolerate food deprivation for at least 45 days, but this tolerance is closely influenced by their previous dietary protein sources. Therefore, it is suggested that animal-sourced protein in the diets of marron can assist in combating any starvation-related stress in marron, particularly during low feed availability.

## Figures and Tables

**Table 1:** Feed formulation and proximate composition.

Ingredients *	Experimental Diets					
	FM	PBM	BSFM	TH	LM	SBM
FM	46.00	0.00	0.00	0.00	0.00	0.00
PBM	0.00	42.00	0.00	0.00	0.00	0.00
BSFM	0.00	0.00	33.60	0.00	0.00	0.00
TH	0.00	0.00	0.00	27.00	0.00	0.00
LM	0.00	0.00	0.00	0.00	70.00	0.00
SBM	0.00	0.00	0.00	0.00	0.00	62.00
Wheat	30.00	34.50	33.40	35.00	7.00	12.00
Corn/wheat starch	11.00	11.00	11.00	11.00	11.00	10.00
Cholesterol	0.50	0.50	0.50	0.50	0.50	0.50
Canola oil	2.00	1.50	0.00	0.00	2.00	4.00
Cod liver oil	3.00	2.00	0.00	0.00	2.50	5.00
Vitamin premix	0.30	0.30	0.30	0.30	0.30	0.30
Vitamin C	0.10	0.10	0.10	0.10	0.10	0.10
Dicalcium phosphate	0.10	0.10	0.10	0.10	0.10	0.10
Lecithin–soy	3.00	3.00	3.00	3.00	3.00	3.00
Barley	4.00	5.00	5.00	4.00	3.50	3.00
Casein	0.00	0.00	13.00	19.00	0.00	0.00
***Proximate composition (%dry weight)***						
Crude protein (%)	30.81	31.06	30.25	31.04	30.06	31.17
Crude lipid (%)	12.99	13.75	13.27	12.32	12.95	12.21
Moisture (%)	8.31	8.23	8.58	7.86	8.50	8.04
Ash (%)	11.07	6.52	4.22	5.17	3.18	5.47

* Fishmeal (FM): crude protein 58.55%, crude lipid 9.46%; black soldier fly meal (BSFM): crude protein 44.04%, crude lipid 28.3%; soybean meal (SBM): crude protein 46.41%, crude lipid 2.51%; lupin meal (LM): crude protein 41.47%, crude lipid 9.01%; poultry by-product meal (PBM): crude protein 62.75%, crude lipid 15.1%; Tuna hydrolysate (TH): crude protein 37.91%, crude lipid 35.50%.

**Table 2:** Amino acid composition (g/100 g dry matter basis) of the test diets.

	FM	PBM	BSFM	TH	LM	SBM
***Essential amino acids (g/100 g on dry matter basis)***						
Histidine	2.95	2.27	2.90	2.82	2.96	2.76
Threonine	4.63	4.03	4.19	4.28	3.98	4.23
Lysine	6.95	5.68	6.61	6.87	4.21	5.61
Arginine	6.25	6.85	4.33	4.35	11.60	7.15
Methionine	2.70	2.05	2.15	2.46	0.61	1.09
Valine	5.51	5.09	6.24	6.04	4.59	5.26
Isoleucine	4.70	4.25	4.98	4.88	4.77	5.04
Leucine	8.18	7.73	8.42	8.53	7.88	8.53
Phenylalanine	4.67	4.36	4.98	4.88	4.62	5.52
***Non-essential amino acids (g/100 g on dry matter basis)***						
**Serine**	4.56	4.43	5.11	5.34	5.53	5.45
Glutamic acid	16.00	17.66	20.04	20.60	22.58	20.21
Glycine	8.14	10.40	3.99	4.41	4.81	4.56
Aspartic acid	9.02	8.06	8.04	7.37	10.16	11.36
Alanine	6.56	6.74	4.70	4.31	3.56	4.52
Proline	6.56	8.02	9.00	9.46	4.96	5.84
Tyrosine	2.63	2.38	4.33	3.42	3.18	2.86

FM: fish meal; PBM: poultry by-product meal; BSFM: black soldier fly meal; TH: tuna hydrolysate; LM: lupin meal; SBM: soybean meal.

**Table 3:** Treatments based on 0^+^marron initially fed on various protein sources before they were exposed to feed deprivation.

	Treatments
SFM	Marron fed initially fish meal.
SPBM	Marron fed initially poultry by-product meal.
SBSFM	Marron fed initially black soldier fly meal.
STH	Marron fed initially tuna hydrolysate.
SLM	Marron fed initially a lupin meal.
SSBM	Marron fed initially soybean meal.

**Table 4:** Growth performance as reflected by WG, SGR, the total number of moults, and the moulting rate of the feed-deprived marron.

Treatments	IBW (g)	FBW (g)	WG (%)	SGR (%/Day)	Total Number of Moults	Moulting Rate (%)
SFM	3.14 ± 0.06	3.32 ± 0.07	8.50 ± 2.26	0.13 ± 0.06	4	13.34 ± 3.34
SPBM	3.01 ± 0.47	3.20 ± 0.56	7.02 ± 1.89	0.14 ± 0.03	3	10.00 ± 5.77
SBSFM	3.48 ± 0.08	3.79 ± 0.11	9.56 ± 2.14	0.18 ± 0.04	4	13.34 ± 8.81
STH	2.86 ± 0.53	3.00 ± 0.58	4.58 ± 0.61	0.09 ± 0.01	4	13.34 ± 3.34
SLM	2.47 ± 0.29	2.60 ± 0.33	5.39 ± 1.35	0.10 ± 0.02	4	13.34 ± 6.67
SSBM	2.83 ± 0.19	2.97 ± 0.13	6.43 ± 1.84	0.13 ± 0.05	4	13.34 ± 8.81
*p* value	0.425	0.369	0.404	0.760		0.999

Values are mean ± SE (n = 3). IBW: initial marron weight, FBW: final marron weight, WG: weight gain, SGR: specific growth rate. SFM: starved marron fed initially fishmeal; SPBM: starved marron fed initially poultry by-product meal; SBSFM: starved marron fed initially black soldier fly meal; STH: starved marron fed initially tuna hydrolysate; SLM: starved marron fed initially lupin meal; SSBM: starved marron fed initially soybean meal.

**Table 5:** Organosomatic indices of starved marron initially fed various diets over 45 days.

Index	Days	Marron Groups					
		SFM	SPBM	SBSFM	STH	SLM	SSBM
HM	0	_1_ 65.09 ± 2.25 ^b^	_1_ 70.45 ± 0.98 ^c^	_1_ 59.20 ± 2.14 ^a^	_1_ 76.00 ± 2.71 ^d^	_1_ 70.89 ± 2.81 ^cd^	_1_ 66.07 ± 1.32 ^c^
	15	_2_ 69.92 ± 2.00 ^b^	_2_ 76.56 ± 2.06 ^c^	_2_ 67.13 ± 1.30 ^a^	_2_ 80.40 ± 2.01 ^d^	_2_ 78.01 ± 1.92 ^cd^	_2_ 78.18 ± 2.03 ^c^
	30	_3_ 77.04 ± 2.21 ^b^	_3_ 79.26 ± 1.09 ^c^	_3_ 63.56 ± 0.59 ^a^	_3_ 83.07 ± 0.44 ^d^	_3_ 83.80 ± 0.98 ^cd^	_3_ 82.11 ± 1.09 ^c^
	45	_3_ 79.98 ± 0.58 ^b^	_3_ 79.93 ± 0.17 ^c^	_3_ 78.21 ± 0.99 ^a^	_3_ 80.41 ± 0.14 ^d^	_3_ 82.36 ± 0.32 ^cd^	_3_ 79.20 ± 0.07 ^c^
TM	0	_1_ 80.60 ± 0.52	_1_ 81.26 ± 0.52	_1_ 79.52 ± 0.66	_1_ 80.91 ± 0.89	_1_ 79.89 ± 0.60	_1_ 80.95 ± 0.47
	15	_2 3_ 82.67 ± 0.65	_2 3_ 82.48 ± 1.23	_2 3_ 81.71 ± 0.95	_2 3_ 82.82 ± 0.87	_2 3_ 83.63 ± 0.25	_2 3_ 83.54 ± 0.29
	30	_3_ 83.25 ± 0.28	_3_ 83.93 ± 0.16	_3_ 82.11 ± 0.53	_3_ 82.73 ± 0.58	_3_ 84.33 ± 0.39	_3_ 83.83 ± 0.11
	45	_2_ 82.65 ± 0.24	_2_ 81.61 ± 0.15	_2_ 82.56 ± 0.12	_2_ 82.19 ± 0.15	_2_ 82.34 ± 0.40	_2_ 82.14 ± 0.57
Hiw	0	_4_ 6.66 ± 0.26 ^abc^	_4_ 6.30 ± 0.20 ^ab^	_4_ 5.77 ± 0.13 ^ab^	_4_ 6.82 ± 0.51 ^bc^	_4_ 6.40 ± 0.26 ^a^	_4_ 6.53 ± 0.32 ^c^
	15	_3_ 4.95 ± 0.18 ^abc^	_3_ 4.31 ± 0.28 ^ab^	_3_ 5.37 ± 0.15 ^ab^	_3_ 4.97 ± 0.07 ^bc^	_3_ 4.11 ± 0.20 ^a^	_3_ 5.67 ± 0.23 ^c^
	30	_2_ 3.69 ± 0.12 ^abc^	_2_ 3.59 ± 0.23 ^ab^	_2_ 3.50 ± 0.13 ^ab^	_2_ 3.54 ± 0.29 ^bc^	_2_ 3.29 ± 0.07 ^a^	_2_ 4.50 ± 0.44 ^c^
	45	_1_ 2.95 ± 0.19 ^abc^	_1_ 3.16 ± 0.05 ^ab^	_1_ 2.51 ± 0.20 ^ab^	_1_ 3.34 ± 0.26 ^bc^	_1_ 2.72 ± 0.08 ^a^	_1_ 3.46 ± 0.18 ^c^
Hid	0	_4_ 2.32 ± 0.09 ^ab^	_4_ 1.86 ± 0.05 ^ab^	_4_ 2.35 ± 0.10 ^c^	_4_ 1.62 ± 0.14 ^a^	_4_ 1.87 ± 0.21 ^ab^	_4_ 2.21 ± 0.09 ^bc^
	15	_3_ 1.01 ± 0.10 ^ab^	_3_ 1.39 ± 0.23 ^ab^	_3_ 1.69 ± 0.24 ^c^	_3_ 1.05 ± 0.07 ^a^	_3_ 1.17 ± 0.24 ^ab^	_3_ 1.22 ± 0.12 ^bc^
	30	_2_ 0.85 ± 0.09 ^ab^	_2_ 0.75 ± 0.08 ^ab^	_2_ 1.28 ± 0.05 ^c^	_2_ 0.61 ± 0.07 ^a^	_2_ 0.53 ± 0.03 ^ab^	_2_ 0.82 ± 0.13 ^bc^
	45	_1_ 0.59 ± 0.05 ^ab^	_1_ 0.64 ± 0.02 ^ab^	_1_ 0.55 ± 0.05 ^c^	_1_ 0.63 ± 0.07 ^a^	_1_ 0.48 ± 0.01 ^ab^	_1_ 0.72 ± 0.04 ^bc^
Tiw	0	_3_ 26.79 ± 0.52	_3_ 27.96 ± 0.96	_3_ 29.82 ± 0.86	_3_ 25.68 ± 0.93	_3_ 27.93 ± 0.98	_3_ 27.79 ± 0.61
	15	_2 3_ 27.16 ± 1.12	_2 3_ 27.56 ± 1.33	_2 3_ 27.41 ± 0.48	_2 3_ 25.38 ± 1.48	_2 3_ 25.84 ± 0.55	_2 3_ 25.52 ± 0.76
	30	_2_ 27.28 ± 0.74	_2_ 26.48 ± 0.83	_2_ 26.89 ± 0.48	_2_ 25.91 ± 0.86	_2_ 25.12 ± 0.22	_2_ 24.51 ± 0.72
	45	_1_ 21.94 ± 0.76	_1_ 24.42 ± 0.77	_1_ 21.56 ± 0.84	_1_ 25.49 ± 0.99	_1_ 24.28 ± 0.71	_1_ 25.12 ± 0.55
Tid	0	_2_ 5.20 ± 0.14	_2_ 5.23 ± 0.07	_2_ 6.10 ± 0.06	_2_ 4.92 ± 0.36	_2_ 5.62 ± 0.22	_2_ 5.30 ± 0.24
	15	_1_ 4.88 ± 0.37	_1_ 4.79 ± 0.81	_1_ 4.93 ± 0.26	_1_ 4.21 ± 0.33	_1_ 4.20 ± 0.13	_1_ 4.55 ± 0.25
	30	_1_ 4.57 ± 0.05	_1_ 4.25 ± 0.09	_1_ 4.81 ± 0.16	_1_ 4.47 ± 0.18	_1_ 3.94 ± 0.12	_1_ 3.97 ± 0.10
	45	_1_ 3.81 ± 0.13	_1_ 4.49 ± 0.15	_1_ 3.77 ± 0.14	_1_ 4.55 ± 0.22	_1_ 4.28 ± 0.03	_1_ 4.49 ± 0.14

Values are mean ± SE (n = 3). Values in the same row with different superscript letters (a, b, c, d) mean significant differences among the diets. Value in the same column with different numerical subscripts (1, 2, 3, 4) denotes significant differences at different periods (two-way ANOVA followed by Tukey post hoc test with *p* < 0.05). SFM: starved marron fed initially fishmeal; SPBM: starved marron fed poultry by-product meal initially; SBSFM: starved marron fed initially black soldier fly meal; STH: starved marron fed tuna hydrolysate initially; SLM: starved marron fed lupin meal initially; SSBM: starved marron fed soybean meal initially. HM: hepatopancreas moisture; TM: tail moisture; Hiw: wet hepatosomatic index; Hid: dry hepatosomatic index; Tiw: wet tail muscle index; Tid: dry tail muscle index.

## Data Availability

The data are contained in this article.
